# Impacts of environmental complexity on respiratory and gut microbiome community structure and diversity in growing pigs

**DOI:** 10.1038/s41598-019-50187-z

**Published:** 2019-09-24

**Authors:** Ameer Megahed, Mohamed Zeineldin, Kaleigh Evans, Nidia Maradiaga, Ben Blair, Brian Aldridge, James Lowe

**Affiliations:** 1 Integrated Food Animal Management System, Department of Veterinary Clinical Medicine, College of Veterinary Medicine, University of Illinois at Urbana-Champaign, Illinois, 61802 USA; 2 Department of Animal Medicine, Internal Medicine, Faculty of Veterinary Medicine, Benha University, Moshtohor-Toukh, Kalyobiya, 13736 Egypt

**Keywords:** Microbiology, Microbiome

## Abstract

The limited understanding of the interaction between rearing environment of the growing pig and the pig’s microbial community impedes efforts to identify the optimal housing system to maximize animal health and production. Accordingly, we characterized the impact of housing complexity on shaping the respiratory and gut microbiota of growing pig. A total of 175 weaned pigs from 25 litters were randomly assigned within liter to either simple slatted-floor (S) or complex straw-based rearing ecosystem (C). Beside the floor swabs samples, fecal swabs and mucosal scraping samples from bronchus, ileum, and colon were collected approximately 164 days post-weaning at the time of slaughter. The S ecosystem seems to increase the α-diversity of respiratory and gut microbiota. Moreover, the C-raised pigs showed 35.4, 89.2, and 60.0% reduction in the *Firmicutes*/*Bacteroidetes* ratio than the S-raised pigs at bronchus, ileum, and colon, respectively. The unfavorable taxa *Psychrobacter, Corynebacterium, Actinobacteria*, and *Neisseria* were the signature taxa of C environment-associated microbial community. Therefore, the microbiota of S-raised pigs seems to show higher density of the most essential and beneficial taxa than the C-raised pigs. We preliminarily conclude that increasing the physical complexity of rearing environment seems to provide suboptimal conditions for establishing a healthy microbial community in the growing pigs.

## Introduction

Our previously pathogenic view of animal–microbe interactions has been changed over the last decade. We now appreciate the essential role of the microbiota in the digestion and absorption of nutrients, prevention of pathogen colonization, maintenance and regulation of normal mucosal immunity and metabolic health, and overall regulation of host physiological functions in association with longevity^1^. The early microbiota colonization and succession is dependent mainly on the maternal and environment-animal or human microbiota exchange^2,3^. The first inoculum for microbiota assembly is maternal-dependent and homogenous across most body sites, however the subsequent progression is environmental-dependent^4^. The environmentally-acquired microbes are therefore essential building blocks of animal microbial community^5^, and any injurious shift in the physical environment can substantially disrupt the composition and functionality of the human and animal microbial community during the growing period^6,7^.

The swine industry is constantly growing and integrating, resulting in different approaches for barn design and production practices. These designs are centered around space, floor, water, drainage, residential housing, existing facilities, manure management, snow and wind control, feed supply, and security^8^. One of the most important environmental factor that has a direct impact on the developmental dynamic of microbial community in pigs is the flooring and bedding system^5,7,9^. In rodents, type of floor drives deleterious changes in the gut microbiota composition and diversity^7,10^. In poultry, rearing on deep letter system causes injurious shifts in gut microbiota structure and functionality^11^. In the pre-weaned piglets, the outdoors-raised piglets exhibited significant reduction in the ileal mucosa-adherent microbial diversity and richness compared to indoors-raised piglets^5,9^. The lack of understanding of the interaction between rearing environments of the growing pigs and their microbial community colonization and succession triggers us to conduct this study. Here, we hypothesized that the growing pigs expose to complex environment during growing period will generate a less diverse unbalanced microbial community. The main objective of this study was therefore to characterize the impact of simple-slatted (S) and complex straw-based (C) rearing environments of the growing pigs on the respiratory (bronchus), gut (ileum and colon) mucosa-associated bacteriome, and gut (fecal) luminal-associated bacteriome structure and diversity in growing pig. The current study offers an extended discussion on understanding the interplay between the environmental complexity and microbiome and exploring the potential long-term impact of these changes on the health and productivity of growing pigs.

## Results

### Body weight

No difference (*P* = 0.564) in the average body weight (BW) was reported between S-raised pigs (99.2 ± 8.1 kg) and C-raised pigs (97.5 ± 8.8 lb) before sending to harvest, with average daily gain 0.9 ± 0.2 and 0.8 ± 0.2 lb, respectively.

### 16S rRNA gene profiles

After trimming and filtering the low quality reads, a total of 120 pig samples (n = 24 pre-exposure; n = 96 post-exposure) and 24 environmental samples were qualified for further data analysis. With a median sequencing depth of 24,746 reads (interquartile range (IQR): 8,030–47,480) for pig samples, we identified 6,688 unique operational taxonomic units (OTUs), demonstrating a median richness of 441 OTUs per sample. The data yielded sequences belonging 20 phyla, 40 classes, 71 Orders, 124 families, and 230 genera, with the dominating genera *Bacteroidetes*, *Clostridium*, and *Streptococcus* (bronchus)*; Helicobacter, Clostridium*, and *Escherichia/Shigella* (ileum); *Clostridium, Prevotella*, and *Streptococcus* (colon); and *Clostridium, Streptococcus*, and *Helicobacter* (feces). In environmental samples, we identified 17,005 unique OTUs, demonstrating a median richness of 3,734 OTUs per sample with a median sequencing depth of 395,630 reads (IQR: 381,114–424,601). The data yielded sequences belonging 15 phyla, 40 classes, 80 Orders, 169 families, and 420 genera, with the dominating genera *Lactobacillus*, *Clostridium*, and *Corynebacterium*.

### Microbial community richness and diversity

**Figure 1:**
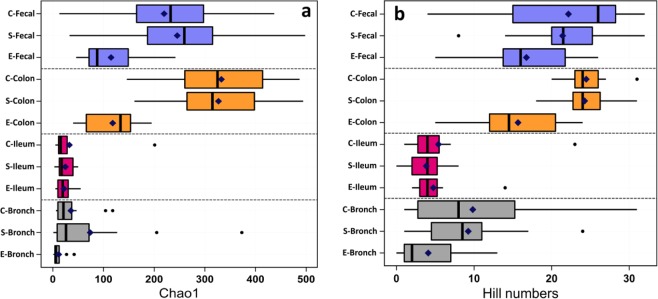
Box and whiskers plots of the alpha-diversity representing in Chao1 richness **(a)** and Hill diversity numbers **(b)** indices of bacteriome at bronchus (bronch), ileum, colon, and fecal in pigs at entry (E) and simple (S) and complex (C) raised- pigs. Dark blue dots indicate mean.

The median and (IQR) of OTUs were observed in the floor, bronchus, ileum, colon, and feces are presented in Table 1. The observed OTUs in the C straw-based floor was higher (*P* < 0.05) than S-slatted floor. Alpha-diversity as assessed by the Chao1 richness and Hill numbers diversity indices are presented in Fig. 1. In bronchus, the Chao1 index showed no difference (*P* = 0.817) in the mucosal microbiota richness between S and C-raised pigs (median (IQR), 26.0 (7.5–108.5) and 21.0 (10.0–43.8), respectively). However, both were significant different (*P* < 0.05) from E-pigs (6.0, 3.0–14.0). Similarly, the Chao1 index showed no difference (*P* > 0.515) in the gut (ileum and colon) mucosal microbiota richness between S and C-raised pigs. However, the differences were found over the age (*P* < 0.01), except the mucosal microbiota of ileum showed no difference between E-pigs and S and C-raised pigs (*P* > 0.525; Fig. 1a).

**Table 1:** The median and interquartile range (IQR) of the operational taxonomic units (OTUs) were observed in the environment, and bronchus, ileum, colon, and feces of pigs at entry and raised on simple-slatted and complex straw-based floors for 20 weeks.

	Entry	Simple	Complex
Environment	7537.9 (7208.9–10430.5)^a^	6672.0 (6053.6–7948.9)^aa^	9450.8 (6358.9–12055.6)^ab^
Bronchus	97.0 (48.7–286.0)	260.9 (81.0–770.4)	226.8 (124.5–469.9)
Ileum	147.9 (88.9–215.9)	152.9 (115.8–330.4)	137.5 (100.4–243.5)
Colon	702.8 (454.0–927.6)^a^	1566.8 (1364.3–1831.7)^b^	1650.7 (1280.6–1966.5)^b^
Fecal	565.3 (481.3–991.4)^a^	1285.7 (1135.0–1591.8)^b^	1280.2 (600.0–1547.6)^b^

^a,b^Values within a row with different letters are significantly different (*P* < 0.05).

In the mucosal microbiota of bronchus and gut, Hill diversity numbers were not different between S and C-raised pigs (Fig. 1b). The Hill diversity numbers of bronchus, ileum, and colon were 8.0 (median, (IQR) 2.3–15.8), 4.0 (2.0–5.8), and 24.0 (22.3–26.8) for S-raised pigs and 8.5 (3.5–13.0), 4.0 (2.3–6.5), and 24.0 (22.0–26.0) for C-raised pigs, respectively. However, in bronchus and colon, the mucosal microbiota diversity of S and C-raised pigs was different (*P* < 0.01) from E-pigs (2.0, 1.0–9.0; 14.0, 12.0–22.0), respectively.

In environmental samples, the complex environment (C-Env; 301.0, 268.0–338.5) showed higher (*P* < 0.001) richness than simple environment (S-Env; 181.0, 165.0–197.0). However, the Hill diversity numbers were not different between S-Env (8.0, 7.0–8.8) and C-Env (10.0, 9.0–11.0).

### The sharing OTUs and dissimilarity percentage between environment and pigs microbiome

For the 1,683 taxonomically annotated OTUs present in the mucosal surface of bronchus of C-raised pigs, 288 (17.1%) were found in their C-Env. However, relatively low level of OTUs shared between bronchial mucosa of S-raised pigs and S-Env, whereas 4,431 OTUs present in S-raised pigs, 527 (11.9%) were found in their S-Env (Fig. S1A). In the mucosal surface of ileum of C-raised pigs, from 1986 OTUs, 377 (19.0%) were found in their C-Env. In contrast, high level of OTUs shared between ileal mucosa of S-raised pigs and S-Env, whereas 1,285 OTUs present in S-raised pigs, 614 (47.8%) were found in their S-Env (Fig. S1B). In the mucosal surface of colon of C-raised pigs, from 14,332 OTUs, 1139 (7.9%) were found in their C-Env. However, relatively high level of OTUs shared between colonic mucosa of S-raised pigs and S-Env, whereas 14,681 OTUs present in S-raised pigs, 1,266 (8.6%) were found in their S-Env (Fig. S1C). In the gut lumen, C-raised pigs have 9,997 OTUs, 1,160 (11.6%) were found in their C-Env. However, relatively high level of OTUs shared between gut lumen of S-raised pigs and S-Env, whereas 11,161 OTUs present in S-raised pigs, 1,116 (10.0%) were found in their S-Env (Fig. S1D). The colon showed the highest OTUs shared with bronchus in both S and C-raised pigs, whereas the shared OTUs between bronchus and colon were 1,230 (73.1%) of 1,683 in C-raised pigs and 3,006 (67.8%) of 4,431 in S-raised pigs.

### Microbial community structure

**Figure 2:**
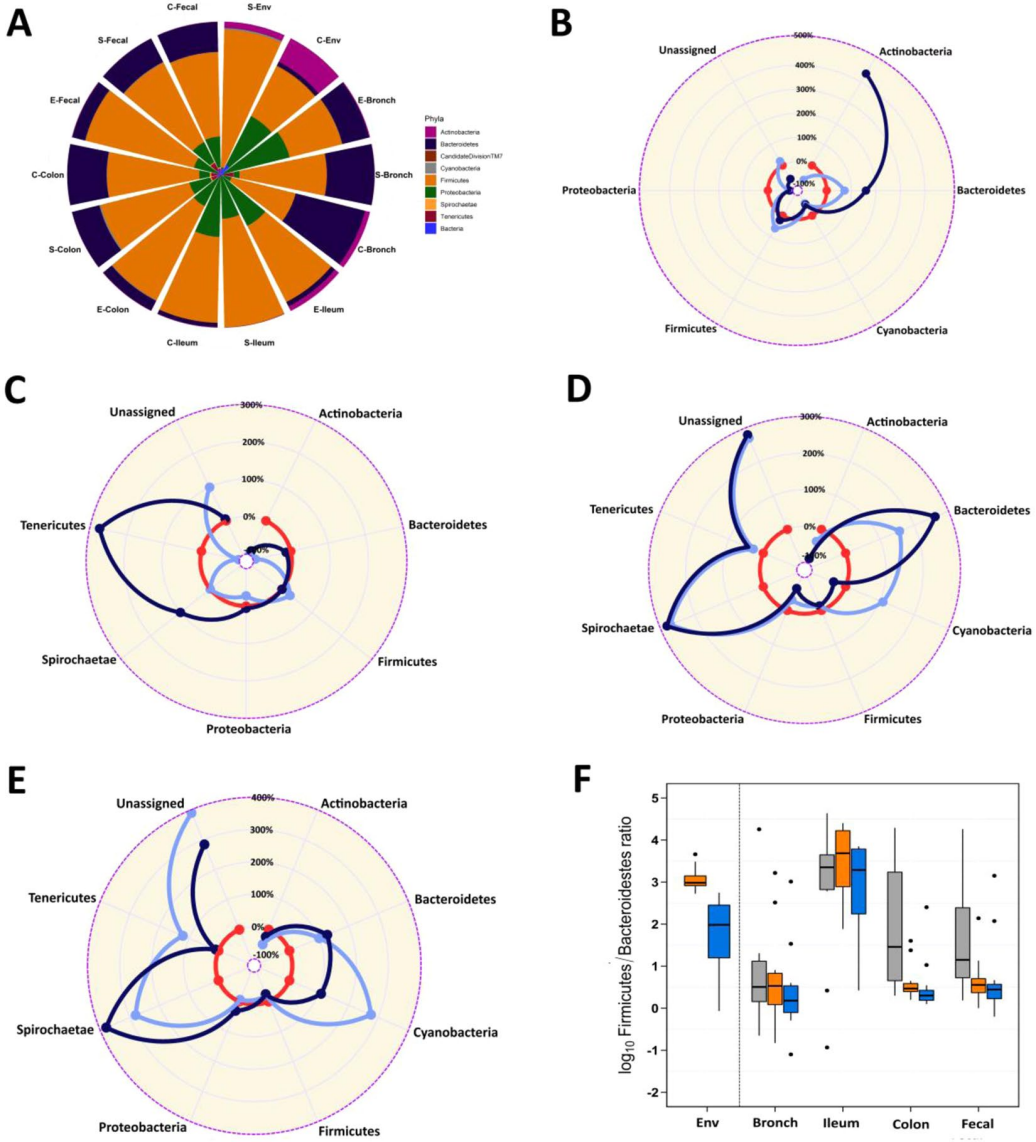
Microbial composition at phylum level. **(A)** Stacked bar chart of relative abundance of the top 9 phyla that explain 99% of the microbiota. **(B)** Spider plot comparing the most abundant phyla at bronchus, ileum **(C)**, colon **(D)**, and fecal (**E)** in pigs at entry (red) and pigs raised in simple (light blue) and complex (blue) ecosystem. **(F)** Box and whiskers plots depicting changes in *Firmicutes/Bacteroidetes* ratios in pigs at E (E, gray), pigs raised in simple (yellow) and complex (blue) ecosystem.

We first determined the changes in the top 9 phyla that explain 99% of the bacterial population composition of S and C-Env, bronchus, ileum, colon, and fecal of pigs at entry and raised on S and C ecosystem (Fig. 2). The most common organisms are members of the gram-positive *Firmicute*s and the gram-negative *Bacteroidetes* phyla, with several others phyla, including the *Proteobacteria, Actinobacteria, and Tenericutes* that are present at subdominant levels. However, *Proteobacteria* was the second most abundant phylum after *Firmicute*s (58.2%) in various selected sites of E-pigs, accounting for 30.0% of the total reads (Fig. 2A). In bronchus, *Actinobacteria* phylum was more abundant (*P* < 0.001) in C-raised pigs compared to S-raised pigs (Fig. 2B). The same result was reported in the environmental samples (Fig. 2A). In ileum, *Bacteroidetes* phylum was more abundant (*P* < 0.001) in C-raised pigs compared to S-raised pigs (Fig. 2C), that was similar to the results of environmental samples. In colon and fecal, no differences was reported between S and C-raised pigs (Fig. 2D,E).

The *Firmicutes*/*Bacteroidetes* (F/B) ratio did not show marked differentiation between S and C-raised pigs. However, in ileum and colon, S-raised pigs seemed to have higher F/B ratio than C-raised pigs. Of interest, the S-Env showed higher F/B ratio than C-Env (*P* ≤ 0.001; Fig. 2F).

**Figure 3:**
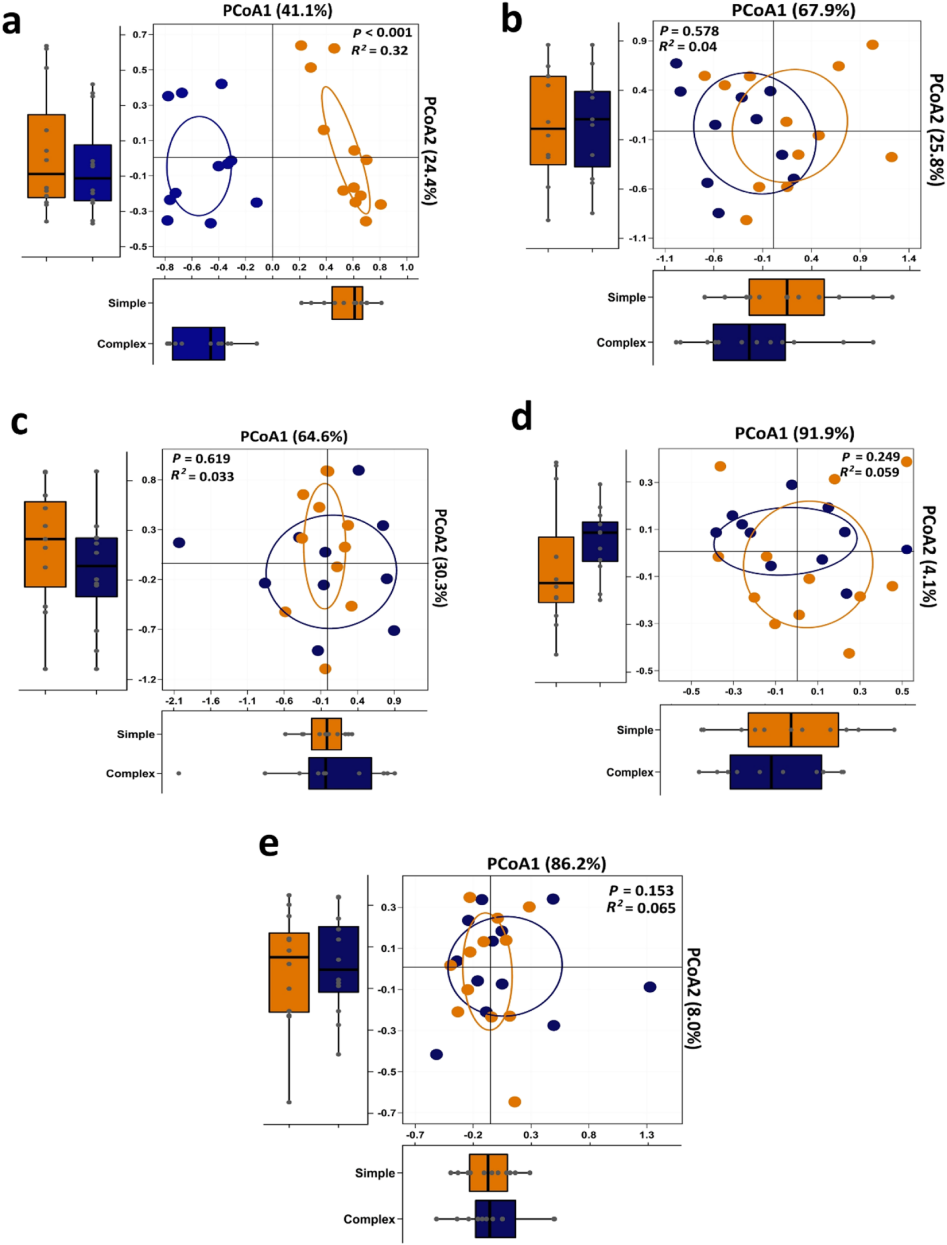
Principle Coordinate Analysis (PCoA) on unweighted UniFrac distances of bacteriome. **(a)** PCoA for simple (yellow) and complex (blue) environments. (**b)** PCoA of mucosal-associated bacteriome at bronchus, ileum **(c)**, colon **(d)**, and PCoA of fecal bacteriome **(e)**. Ellipses represent a 95% CI around the cluster centroid. Box-and-whisker plots shown along each PCoA axis indicate the distribution of samples along the given axis.

Pairwise Bray–Curtis dissimilarity comparisons (beta diversity) showed marked differentiation (Adonis *P* < 0.001) in the bacterial community structure between S and C-Env, with R^2^ value of 0.30 (Fig. 3a). However, no marked differentiations (Adonis *P* > 0.05) in the bacterial population were reported between S and C-raised pigs at bronchus (Fig. 3b), ileum (Fig. 3c), colon (Fig. 3d), and fecal (Fig. 3e).

The linear discriminant analysis (LDA) analysis showed several characteristic taxa for the S and C-Env as presented in Fig. S2A. In bronchus, the most discriminating indicator taxa for mucosal-associated microbiota of S-raised pigs is *Clostridium*. In ileum and fecal, the LDA did not identify any characteristic taxa for bacterial population. However, several signature taxa have been reported for colonic mucosa-associated microbiota in S and C-raised pigs (Fig. S2B).

**Figure 4:**
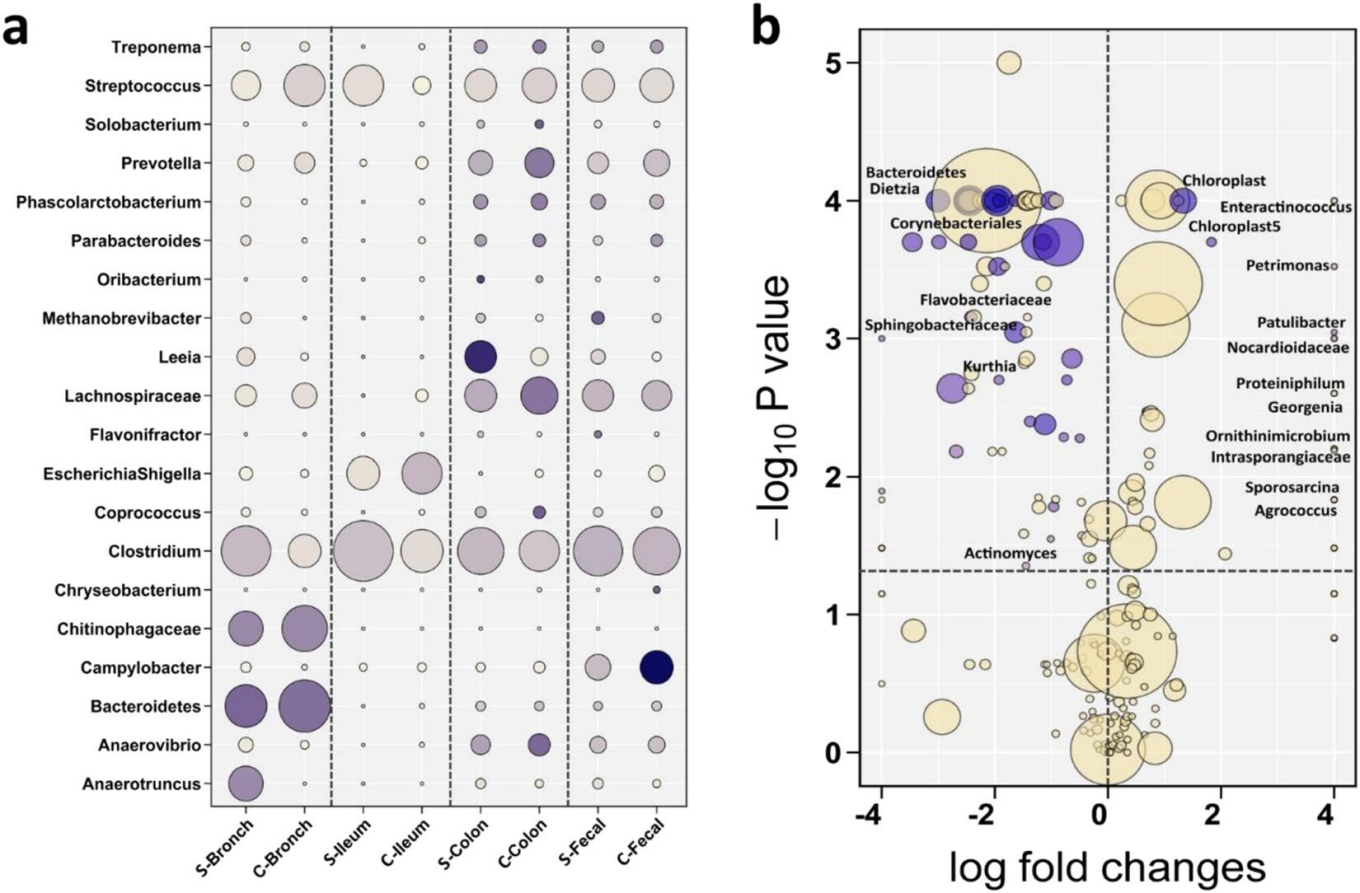
Microbial composition at genus level. **(a)** Bubble plot of the average relative abundance (circle size) of the most prevalent genera (y axis) of bronchial, ileum, colon, and fecal microbiota in pigs raised on simple slatted and complex straw-based floors. The indicator value index (IndVal; related to the shading of circle color) reflects the strength of association between genera and a given group. Darker color indicating larger values that reflecting greater specificity. **(b)** Bubble plot showing the significance differences between the simple and complex environment at genus level. X-axis reports the log_2_ ratio (simple/complex) of the relative abundances; Y-axis depicts the -log_10_(*P*-value) of the two-sided Mann-Whitney U-test for comparing bacterial groups; bubble dimension is related to the average relative abundance of taxa. Shading of circle color indicating IndVal. Bacterial groups namely indicated example of the signature core taxa of the environment types.

The indicator value index (IndVal) results indicated that *Anaerotruncus* was considered a signature taxon for bronchial microbiota of S-raised pigs (IndVal = 0.22; *P* = 0.01), with average relative abundance (RA) of 8.0%, and *Bacteroidetes* and *Chitinophagaceae* were considered signature taxa for bronchial microbiota of C-raised pigs (IndVal = 0.29, 0.22; *P* = 0.02), with average RA of 24.3 and 15.4%, respectively. In ileum, *Clostridium* was a signature taxon (IndVal = 0.15; *P* < 0.05) for S-raised pigs and *Escherichia/Shigella* (IndVal = 0.18; *P* < 0.01) for C-raised pigs, with RA of 30.0 and 12.0%, respectively. In colon, *Leeia* and *Clostridium* were signature taxa (IndVal = 0.44, 0.18; *P* < 0.05) for S-raised pigs with RA of 7.0 and 15.0%, respectively. However, *Solobacterium*, *Coprococcus, Anaerovibrio*, *Lachnospiraceae*, *Prevotella*, and *Treponema* were signature taxa (IndVal = 0.31, 0.31, 0.29, 0.26, 0.25, 0.24; *P* < 0.01) for C-raised pigs with RA of 0.2, 0.7, 3.0, 10.0, 6.0, and 0.8%, respectively. In fecal samples, *Methanobrevibacter* and *Flavonifractor* were signature taxa (IndVal = 0.30, 0.25; *P* < 0.01) for S-raised pigs with RA of 0.8 and 0.1%, and *Campylobacter* and *Chryseobacterium* (IndVal = 0.50, 0.3; *P* < 0.01) for C-raised pigs with RA of 8.0% and 0.1%, respectively (Fig. 4a).

The dominant taxa for microbiota of S and C-Env are presented in Table S1 and Fig. 4b. the C-Env showed bigger core microbial community represented by high IndVal than S-Env. *Enteractinococcus*, *Aeromicrobium*, *Chloroplast* taxa were present mainly in S-Env with IndVal of 1.0. However, *Bacteroidetes, Dietzia, CandidateDivisionTM7, Brachybacterium, Jeotgalicoccus, and Gulosibacter* were present mainly in C-Env with IndVal of 1.0.

**Figure 5:**
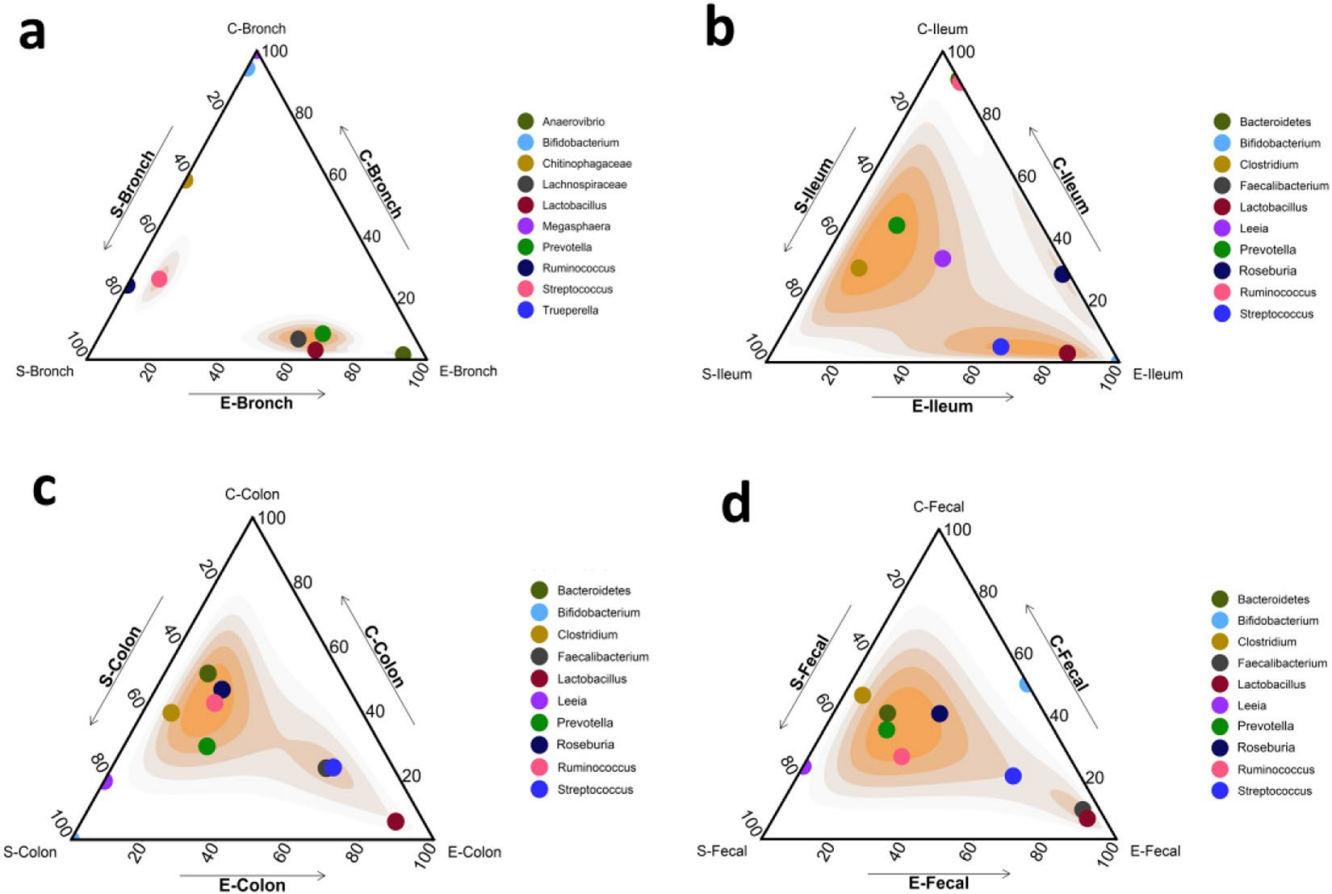
The proportion and density of the most 10 essential beneficial taxa. **(a)** Ternary plot of the most 10 essential beneficial taxa at bronchial, ileal **(b)**, colonic mucosa-associated bacteriome **(c)**, and fecal luminal-associated bacteriome (**d)** of pigs at entry (E), and simple (S) and complex (C)-raised pigs. Axes indicate relative factor density. Color scale indicates concentration of taxa.

The results of ternary plots revealed that the S-raised pigs microbiota showed higher density of the 10 most essential and beneficial taxa at bronchus (Fig. 5a), ileum (Fig. 5b), colon (Fig. 5c), and fecal (Fig. 5d), than the C-raised pigs.

## Discussion

With the primary objective of investigating the impact of housing complexity on the microbial community structure and diversity in growing pigs, we found that the simple-slatted flooring system seems to provide optimal environmental conditions for establishing balanced microbial communities harboring the respiratory tract and gut of growing pigs. To the best of our knowledge, this is the first study characterized the interplay between the flooring systems commonly used in the swine industry and the microbial community structure and diversity in growing pigs. The major limitation of this study was the one time-point sampling at the end of the study period, therefore further studies should be conducted to determine the impact of flooring system on the developmental changes in microbiota over the finishing period in growing pigs. We failed to identify marked differences between S and C-raised pigs related to bacterial population structure and diversity and this is might be due to small sample size of animals and inter-individual variations in the microbial composition and diversity. Additionally, the standard approaches were only used for data analysis instead of compositional approaches. Therefore, the metagenomic information revealed in this study represents only one-step to understand the impact of environmental complexity on the growing pig’s microbiota.

Our finding of numerical increase in the BW of S-raised pigs than the C-raised pigs was inconsistent with the results of earlier pig studies that reported increased feed intake and growth performance of pre-weaned piglets housed with C-Env exposure or outdoor access^12–14^. These studies explained, in part, this increase in the BW to the highly diverse environmental-associated microbiota that harbored the C-Env and has an important role in optimizing the gut function^15^. Additionally, the complex flooring system containing soil provides animals with both plant-derived compounds (carbohydrates, fibers) and soil-borne beneficial microorganisms^16^. However, the C-Env especially the unhygienic C-Env is also an attractive medium to the harmful taxa. These taxa cause deleterious shifts in the normal gut microbiota composition and diversity^17^. This fact might explain the numerical decrease in the growth performance of C-raised pigs.

Typically, the maternal microbiota is considered the main source for establishing the microbial community of their offspring^18^. However, the environmental modulators play also a critical role in the microbiota colonization and succession during the early and subsequent stages of life^19^. Therefore, the diversity of pig’s microbiota relies on the composition and dispersion of maternal and environmental microbial community^5^. Despite the C-raised pigs in this study exposed to highly diverse environmental microbiota, which is biologically sensible because the vulnerability of straw-based floor to constant pressures including humidity and temperature changes^20^, S-raised pigs possessed a higher microbiota diversity. This finding is considered somewhat surprising outcome, because the exposure to C-Env should provide positive potential on the respiratory and gut microbiota in term of increasing the richness and evenness^5^. Interestingly, similar finding has been reported earlier in the pre-weaned piglets, where the early-life piglets raised outdoors and exposed to large variety of environmental microbes showed lower gut microbiota diversity compared to piglets raised indoors^5,9^. This could be explained by prevailing of harmful taxa within the such unhygienic C-Env. Part of these harmful taxa moves to animal microbial community and decreases the opportunity for new beneficial bacteria to inhabit or flourish within microbiota and consequently impede the amplification of microbial diversity^17^. There is substantive evidence that the reduction in the microbial diversity makes the gut epithelium more vulnerable to pathogens through degradation of glycans, which compose the mucus layer that protecting the gut epithelium from pathogens invasion^21,22^. Moreover, the degradation of mucus glycans can release sugars, such as fucose, galactose, or mannose, which can promote the growth of pathogens^23^.

Most of bacterial phylotypes identified in this study had been identified in human and different animal species studies^5,24–26^. The most notable differences between S and C-raised pigs microbiota were reported in the bronchial and ileal mucosal-associated microbiota. These differences included enrichment in *Actinobacteria* and *Bacteroidetes* sequences in bronchial and mucosal-associated microbiota, and *Tenericutes, Spirochaetae*, and *Bacteroidetes* sequences in ileal mucosal-associated microbiota of C-raised pigs in agreement with the earlier pig studies^5,9^. Intriguingly, the same signature phylotypes (*Actinobacteria*, *Bacteroidetes*, and *Proteobacteria*) have been also reported in the C-Env. These signature taxa were also reported in the outdoor C-Env^5^, and deep and wet litter system in poultry^11,27^. *Firmicutes* phylum in this study was higher in the S-raised pigs than the C-raised pigs in contrast with the earlier pig studies^5,9^, that reported a significant increase in the *Firmicutes* phylum in the piglets housed in the outdoor C-Env. Interestingly, the most abundant taxa of S-Env are also members of *Firmicutes* phylum. This provides a strong evidence of sharing a great amount of pig-microbiota with the environment^28^.

The *Firmicutes*/*Bacteroidetes* ratio can provide a useful indicator about the overall gut microbiota balance^29^. The *Firmicutes* phylum includes more than 250 genera, such as *Lactobacillus* and *Clostridium*, while *Bacteroidete*s includes around 20 genera, mostly *Bacteroides*^30,31^. The members of *Firmicutes* phylum are very important for producing short-chain fatty acids (SCFAs) and regulating systemic immune responses. Thus, they are likely involved in maintaining energy balance, inhibiting opportunistic pathogens, and protecting the host against excessive intestinal inflammation^32,33^. In pigs, the increase in the F/B ratio as reported in the S-raised pigs is mostly associated with significant increase in the energy harvest and fat deposition^34^. Therefore, the simple slatted-floor system seems to provide optimal conditions for healthy core native microbiota during the finishing period in pigs.

The different analysis methods were complementary and provided information about the particular taxa that differentiate between the S and C-raised pigs, and environment-associated microbiota. In bronchus, the S-raised pigs were dominated by genus *Anaerotruncus* that is considered one of 17 strains induce a suppressive environment for inducing regulatory T-cells that modulate the immune system, maintain tolerance to self-antigens, and prevent autoimmune disease^35,36^. Additionally, these strains have also been associated with improved outcomes from fecal transplant to treat inflammatory bowel disease in humans^37^. However, genus *Escherichia/Shigella* was the signature taxa of ileal mucosa-associated microbiota of C-raised pigs. Many studies have been reported that the increase in *Escherichia/Shigella* abundance associated with inflammatory bowel disease in humans and pigs^38,39^. The microbial community of diarrheal piglets and growing pigs with necrotizing enterocolitis is dominated by genus *Escherichia/Shigella*^40–43^. The results of this study showed that the *Leeia* genus was the dominant taxa of the colonic mucosa-associated microbiota in S-raised pigs. *Leeia* species play an important role in fermenting the resistant starch in pigs, which is the fraction of ingested starch that escapes enzymatic digestion by endogenous enzymes in the upper gastrointestinal tract and passes into the cecum and colon, where it can be fermented by cecal and colonic microbes^44–46^. Of interest, *Campylobacter* was the dominant taxa of luminal-associated microbiota in the C-raised pigs, which is considered the most common cause of human bacterial enteritis in the developed countries^47^. Most of *Campylobacter* species have a pathogenic role in the development of piglet diarrhea, where they are associated with the intestinal inflammatory disorders and diarrhea in piglets^48,49^. However, it is worth noting at this point that not all members of these unbeneficial taxa lead to health problems. The harmful taxa need to overwhelm the microbial community to cause illness^50^. The relative increases in these unfavorable groups might cause a reduction in the respiratory tract and gut health and function and work also as a stress factor for the host immune system. These might reflect on the animal performance inform of reduction in production or causing subclinical illness^51^.

Another interesting finding is that the C-Env microbial community was dominated with unfavorable taxa, such as *Corynebacterium, Actinobacteria*, and *Neisseria* that are mostly associated with bacteraemia, pulmonary infections, and diarrhea in humans and pigs^48,52^. The member of theses genera are also a major concern because their ability to develop resistance to a wide range of antimicrobials^53^. These observations indicate that the unfavorable taxa under unhygienic C-Env maintain the competition with the beneficial members of microbiota in the growing pigs as shown in Fig. 5, while the S-raised pigs showed higher density of the most essential taxa than the C-raised pigs. *Bifidobacterium*, *Chitinophagaceae, Streptococcus*, and *Ruminococcus* are considered important members of the healthy lung microbiota because their role in intensifying the lower respiratory tract defense mechanism against the colonization of pathogens^36,53^. In gut, *Bifidobacterium*, *Leeia*, *Prevotella*, *Roseburia*, and *Ruminococcus* are essential taxa for metabolizing wide range of complex oligosaccharides and polysaccharides and production of SCFAs. The members of these taxa facilitate the breakdown of proteins and carbohydrates in feed. Furthermore, they have an anti-inflammatory regulation of the host^3,54^.

## Conclusions

Based on the standard approaches used in this study for microbiome data analysis, the simple slatted-floor seems to provide optimal conditions for colonization and succession of balanced microbial community in growing pigs, which probably has potential long-term impacts on the pig health and productivity.

## Methods

All methods were evaluated and approved by the University of Illinois Institutional Animal Care and Use Committee (IACUC Protocol No.: 14,288). All experiments were performed in accordance with relevant guidelines and regulations.

### Animal and experimental design

A convenience sample of 175 (19–22 day old) commercial genotype (YxL) weaned piglets from 25 litters were enrolled in the study between May 1, 2014 and May 15, 2015. Six piglets (4 gilts, 2 barrows) from each litter were selected. One additional barrow was selected from each litter at entry (E) to serve as a baseline reference for the microbiome at the beginning of the experiment for each litter. Piglets were managed in 5 batches spaced 4–10 weeks apart depending on availability from the source. Each batch included 5 litters (n = 35; 7 × 5). Piglets were commercially reared under controlled environment free of major endemic diseases such as porcine reproductive & respiratory syndrome, porcine epidemic diarrhea virus, *Mycoplasma hyopneumoniae*, *Actinobacillus pleuropneumoniae* prior to move to the Veterinary Medical Research Farm (VMRF) at University of Illinois at Urbana-Champaign. At weaning, piglets were placed in a slatted floor conventional nursery at the VMRF with approximately 3.0 sq.ft per head for 24 days. In a randomized complete block design, 1 barrow and 2 gilts from each litter were then randomly assigned to one of two types of rearing ecosystem, S (n = 15/batch) and C (n = 15/batch). At that time, one barrow from each litter was sacrificed as a baseline reference for microbiome. Pigs were stayed in both ecosystems until 164 days post-weaning. Pigs within both ecosystems were fed consistent commercial diets adjusted for age to meet NRC requirements.

S-raised pigs (n = 75) were maintained on totally slatted floors in temperature-controlled rearing ecosystem. Pigs were housed in 5 pens of 3 pigs each (1 litter per pen) and allotted >12 sq. ft. per head. In the first 24 days, pigs were housed on plastic slatted floors (grower room). The remaining 16 weeks, pigs were transferred to concrete slatted floor (finisher room). Pens were washed weekly with low pressure high volume water. For the complex straw-based ecosystem, pigs (n = 75) were remained in the same pen for a period of 20 weeks. Pens were bedded as needed to maintain a dry place to sleep. The bedding was kept undisturbed during the growing period and gross manure was removed from the dunging area weekly to promote health and control ammonia production. All pens were washed, inspected, disinfected, and air-dried prior the introduction of the next batch.

### Biosecurity protocols

Biosecurity protocols were established for the staff entering each specific ecosystem. For instance, all workers met in neutral building upon their arrival. This safe zone was located between the S and C ecosystems outside the security premise. In this building, personnel changed into basic work overalls and boots. Once removing themselves from the safe zone, they were not allowed back inside until all S samples were collected. White hazmat suits were placed over overalls for ultimate hygienic cleanliness upon emittance to S-raised pigs. Workers used the foot bath sanitizing method upon entering and leaving the S ecosystem. After initial boot sanitation, each employee was required to place plastic booties on top of their existing footwear in order to enter the barn. Gloves were obligatory and used between pens and batches or as needed. After samples collection was completed, the used biosecurity suits, booties, and contaminated gloves were thrown away.

For entering the complex environment, workers were allowed to return to the safe zone to store collected samples and grab the necessary equipment for complex samples collection. Biosecurity traffic control patterns prevented anyone from returning to the simple environment after making trip to the complex environment. The same biosecurity protocols as described for S ecosystem were used for entering the complex ecosystem. After use, all equipment was bleached, washed, and air-dried.

### Sample collection

For ante-mortem sampling, BW was measured at 0, 24, 52, 108, and 164 days post-weaning. Pigs were chalked and humanely ushered into livestock caged weight scanners in order to receive an accurate mass measurement. The luminal microbiome was investigated by taking deep fecal swabs at 164 days post-weaning. Pigs were humanely restrained using a snare and a mouth gag in order to collect deep fecal swabs (Pur-Wraps^®^, Puritan Medical Products, Gulford, Maine). For post-mortem sampling, one barrow per litter per treatment was sent to harvest at 164 days post-weaning. At harvest, mucosal scrapings of bronchus, ileum, and colon were performed. For gut samples, a clamping technique on the esophagus and rectum was applied to prevent gastrointestinal spillage and intestinal contamination. Sterile surgical instruments were used to maneuver around the gastrointestinal tract and delicately remove part of ileum and colon. At each selected site, the dissected parts were immediately put into a petri-dish, where they were washed several times with sterile phosphate-buffered saline. This solution allows for the removal of free floating bacterial and aids in the collection of mucosal scraping content. This mucosal content was aseptically scraped off with #20 surgical blade.

Three floor swabs were also collected per batch within each ecosystem at 0, 24, 52, 108, and 164 days. Swab samples were taken randomly over the entire surface area of the ecosystem of interest using sterile swabs (Pur-Wraps^®^, Puritan Medical Products, Gulford, Maine). All samples were collected and temporarily stored on ice until stored later at −20 °C for DNA extraction.

### DNA Isolation

In a decontaminated, sterile environment, total genomic DNA was extracted from each fecal swab using the power^®^ Fecal DNA isolation Kit (MO BIO Laboratories, Inc., Carlsbad, CA) using the manufacturer’s standard protocol^26^. For each sample, the total DNA concentration and integrity was evaluated by optical density using a Nanodrop™ spectrophotometer (NanoDrop Technologies, Rockland, DE, USA) at wavelengths of 260 and 280 nm, and agarose gel electrophoresis (Bio-Rad Laboratories, Inc, Hercules, CA, USA). The extracted nucleic acids (NAs) were immediately stored at −20 °C pending sequencing the gene encoding the 16S rRNA.

### Sequencing analysis for the 16S rRNA gene amplification

At the DNA Services lab, W. M. Keck Center for Comparative and Functional Genomics (University of Illinois at Urbana-Champaign, Urbana, IL, USA), the quality of total DNA in each sample were re-evaluated on a Qubit™ fluorometer (Life technologies, Grand Island, NY, USA) using the High Sensitivity DNA Kit (Agilent Technologies, Santa Clara, CA, USA). To characterize the microbial composition of each sample, the 16S rRNA genes were amplified from the extracted DNA on two PCR steps. In the first PCR reaction, the V3–V4 hypervariable region of the gene encoding the 16S rRNA was amplified using the modified broad range primers V3-F357 (5′-CCTACGGGAGGCAGCAG-3′) and V4-806R (5′-GGACTACNVGGGTWTCTAAT-3′) on a Fluidigm Biomark HD™ PCR machine (Fluidigm Corporation, South San Francisco, CA, USA) using the default Access Array cycling program without imaging. Harvested products were quantified using the QuantiT™ PicoGreen^®^ fluorometer (Life Technologies, Grand Island, NY, USA) and stored at −20 °C. In the second PCR reaction, sequencing primers and adaptors were added to the amplicon products.

The amplicon regions were quantified using a Fragment Analyzer™ (Advanced Analytics, Ames, IA, USA). PCR products were cleaned and size selected on a 2% agarose E-gel™ (Life Technologies, Grand Island, NY, USA) and extracted from the isolated gel slice with the QIAquick Gel extraction kit (Qiagen, Valencia, CA, USA). To confirm the optimal profile, cleaned, size-selected product was examined on an Agilent Bioanalyzer™. Amplicon sequencing was performed using the Illumina MiSeq™ platform (Illumina, San Diego, CA, USA) according to the manufacturer’s instructions.

The sequence run generated Illumina base call (bcl) files were converted into demultiplexed, compressed fastq files using bcl2fastq 1.8.4 (Illumina, CA). A secondary pipeline was used to decompress the fastq files, generate quality score plots (FastX Tool Kit), and to report the number of reads per sample per library. The bcl files were also processed in bcl2fastq 1.8.4 without demultiplexing, and then sorted by initial PCR-specific primer using a custom in-house pipeline. After preprocessing, the raw sequence data was filtered to remove low quality reads.

#### Data processing

The raw sequence data (fastq-files) for the 16S-rRNA-encoding gene was processed using the open-source software package, Quantitative Insights Into Microbial Ecology platform (QIIME®; v. 1.9.0), using the default parameters on the suggested pipeline (http://qiime.org/tutorials/tutorial.html) with notable changes as described below^55^. Sequences were filtered to remove sequences less than 200 nt, greater than 1,000 nt or with a minimum average quality score (Phred) less than 25, maximum number of ambiguous bases equal 6 and homopolymer runs of >6 bp^56^. Chimeric sequences were identified and removed using UCHIME^57^. All denoised sequences were clustered and de novo operational taxonomic units (OTUs) were identified. Sequences were aggregated into distinct OTUs at 97% similarity using the UCLUST method^58^. To assign taxonomy for each OUT, sequences were retrained against SILVA taxonomic database (http://www.arb-silva.de). The QIIME^®^ software was then used to calculate alpha diversity indices (an estimate of bacterial community richness in a sample) using the Chao1 after all samples have been randomly rarefied to 1,000 sequences per pig sample and 10,000 sequences per environment sample using QIIME software.

#### Statistics and data analysis

To handle the microbiome data, RStudio (version 1.1.383, R Studio, Inc., Boston, MA, USA) and SAS 9.4 statistical softwares (SAS Inc, Cary, NC) were used. Significance was determined by Mann–Whitney U tests and mixed models analysis of variance with an autoregressive covariance structure using PROC MIXED. Data were expressed as median and interquartile range (IQR), and *P* < 0.05 was considered significant. Venn diagrams were created for graphical descriptions of the number of unique and shared bacterial OTUs using VennDiagram package under RStudio software. A rough estimation of the Firmicut F/B ratio was obtained by dividing the total number of reads assigned to the Firmicutes phylum by the total number of reads assigned to the Bacteroidetes phylum. Diversity within samples (alpha diversity) and between samples (beta diversity) were calculated. For alpha diversity, Hill numbers (or effective numbers of species) was calculated to control the contributions of rare taxa on determining diversity as follow:

$${}^{q}D={(\mathop{\sum }\limits_{i=1}^{s}p{}_{i}{}^{q})}^{1/(1-q)}$$

where *D* is the diversity, *S* is the number of species, *p* is the species abundance proportions, and *q* is the order number of diversity^59,60^. For beta diversity, weighted UniFrac, Bray–Curtis, and Jaccard distances were determined after adding a pseudocount (+1) to all OTU counts and log-trans forming the OTU tables^61^. Differences in beta diversity between groups at different time points were displayed with principal coordinates analysis (PCoA) plots, in which the significance difference between clustering was determined using a permutational multivariate analysis of variance (PERMANOVA) by Adonis from the package vegan with 999 permutations^62,63^. *P*-values were adjusted for multiple hypothesis testing using the Benjamini-Hochberg procedure (false discovery rate [FDR] = 0.05). To identify the taxon showing significant differences between groups at different time points, Linear discriminant analysis effect size (LEfSe v. 1.0) was performed with a LDA cut-off of 2.0, with a significance value (*P*) of 0.05^64^, through the Galaxy framework^65,66^. To measure the specificity of a taxon to a given biogeographic location at certain group, we determined its indicator value index (IndVal), which considers the relative abundance (RA) of a taxon in a given biogeographic location at certain group and its relative frequency of occurrence across all animals in this group^67^. Ternary plots were also constructed to compare the concentration and density distribution of the most 10 essential, core, and beneficial taxa^25,36,43,46^, between pigs at E and S and C-raised pigs^68^.

### Ethics approval and consent to participate

This study was approved by the the University of Illinois Institutional Animal Care and Use Committee (IACUC Protocol No.: 14288).

## Supplementary information

Supplementry Information

## Data Availability

Fastq data obtained in the current study were publically available on sequence read archive (SRA), National Center for Biotechnology Information (NCBI) website (http://www.ncbi.nlm.nih.gov/sra) with bio-project accession numbers PRJNA515035 and PRJNA508520.
