# Supplementary material for: Impacts of environmental complexity on respiratory and gut microbiome community structure and diversity in growing pigs
Source: Sci Rep. 2019 Sep 24;9:13773. doi: 10.1038/s41598-019-50187-z (PMC6760116; doi:10.1038/s41598-019-50187-z)
Supplement: Supplementary file 1 — Supplementry Information [file 41598_2019_50187_MOESM1_ESM.docx]

**Impacts of environmental complexity on respiratory and gut microbiome community structure and diversity in growing pigs**

Ameer Megahed^1,2^, Mohamed Zeineldin^1,2^, Kaleigh Evans^1^, Nidia Maradiaga^1^, Ben Blair^1^, Brian Aldridge^1^, James Lowe^1,*^

^1^Integrated Food Animal Management System, Department of Veterinary Clinical Medicine, College of Veterinary Medicine, University of Illinois at Urbana-Champaign, Illinois, 61802, USA.

^2^Department of Animal Medicine, Internal Medicine, Faculty of Veterinary Medicine, Benha University, Moshtohor-Toukh, Kalyobiya, 13736, Egypt.

*corresponding. jlowe@illinois.edu

**Figure S1.** Venn diagram describes the number of unique and shared OTUs between the environment (Env) and pigs at the mucosal surface of (a) bronchus (Bronch), (b) ileum, and (c) colon, and (d) gut lumen (feces).


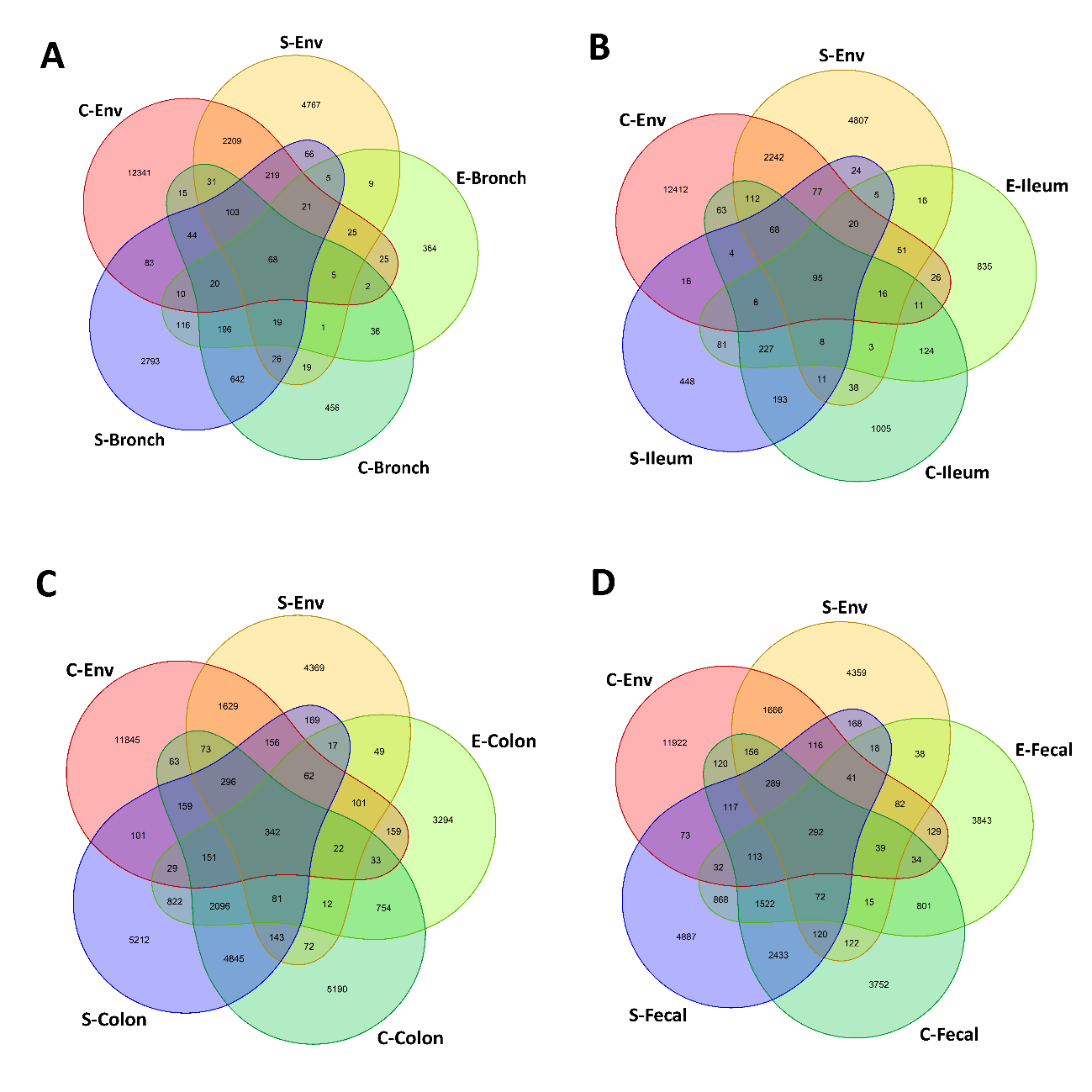


**Figure S2.** Characteristics taxa of the environmental microbial community (a) and colon mucosal microbiota (b) at by Linear Discriminant Analysis Effect Size (LEfSe). Linear Discriminant Analysis (LDA) Score < 5, *P* < 0.05 are shown.


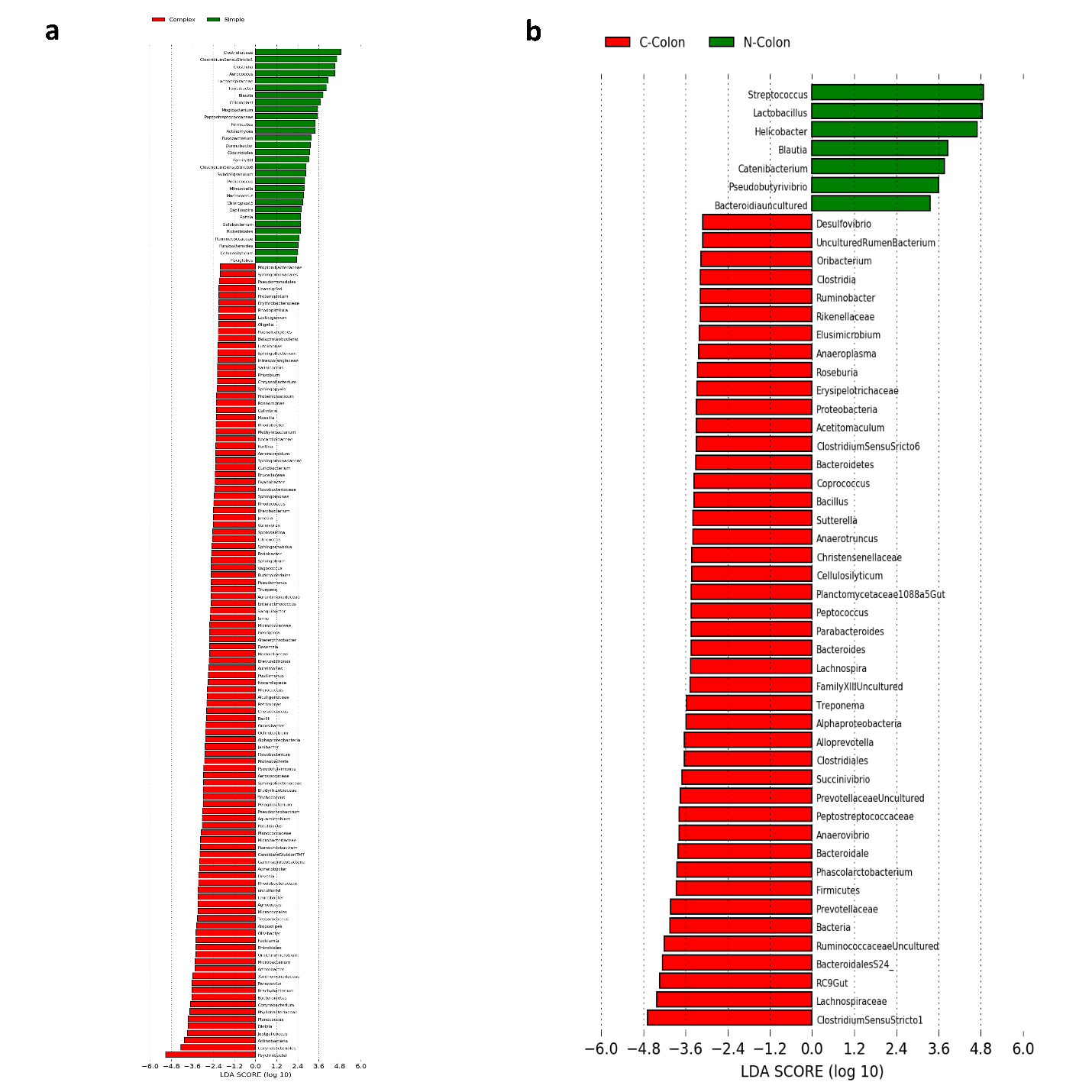


**Table 1S.** The average relative abundance (RA) and indicator value index (IndVal) of the most prevalent genera in simple-slatted and complex straw-based floors. The IndVal reflects the strength of association between genera and a given group. Larg values reflect greater specificity.

| **Genus** | **Group** | **RA** | **IndVal** | **P-value** |
| --- | --- | --- | --- | --- |
| **Enteractinococcus** | Simple | 0.0 | 1.0 | 0.0001 |
| **Aeromicrobium** | Simple | 0.0 | 1.0 | 0.0001 |
| **Chloroplast** | Simple | 0.0 | 1.0 | 0.0001 |
| **Citricoccus** | Simple | 0.0 | 0.9 | 0.0001 |
| **Flavobacterium** | Simple | 0.0 | 0.9 | 0.0001 |
| **Curtobacterium** | Simple | 0.0 | 0.8 | 0.0001 |
| **Chloroplast5** | Simple | 0.0 | 0.8 | 0.0002 |
| **Petrimonas** | Simple | 0.0 | 0.8 | 0.0003 |
| **Parabacteroides** | Simple | 0.0 | 0.7 | 0.0034 |
| **Nocardioidaceae** | Simple | 0.0 | 0.7 | 0.001 |
| **Patulibacter** | Simple | 0.0 | 0.7 | 0.0009 |
| **Truepera** | Simple | 0.0 | 0.7 | 0.001 |
| **Georgenia** | Simple | 0.0 | 0.6 | 0.0025 |
| **Proteiniphilum** | Simple | 0.0 | 0.6 | 0.0025 |
| **Ornithinimicrobium** | Simple | 0.0 | 0.5 | 0.0063 |
| **Intrasporangiaceae** | Simple | 0.0 | 0.5 | 0.0063 |
| **Agrococcus** | Simple | 0.0 | 0.4 | 0.0146 |
| **Sporosarcina** | Simple | 0.0 | 0.4 | 0.0147 |
| **Bacteroidetes** | Complex | 0.0 | 1.0 | 0.0001 |
| **Dietzia** | Complex | 0.0 | 1.0 | 0.0001 |
| **CandidateDivisionTM7** | Complex | 0.0 | 1.0 | 0.0001 |
| **Brachybacterium** | Complex | 0.0 | 1.0 | 0.0001 |
| **Jeotgalicoccus** | Complex | 0.0 | 1.0 | 0.0001 |
| **Gulosibacter** | Complex | 0.0 | 1.0 | 0.0001 |
| **Actinobacteria** | Complex | 0.0 | 0.9 | 0.0002 |
| **Microbacterium** | Complex | 0.0 | 0.9 | 0.0042 |
| **Microbacteriaceae** | Complex | 0.0 | 0.9 | 0.0002 |
| **Leucobacter** | Complex | 0.0 | 0.9 | 0.0001 |
| **Planococcaceae** | Complex | 0.0 | 0.9 | 0.0002 |
| **Tessaracoccus** | Complex | 0.0 | 0.9 | 0.0003 |
| **Corynebacteriales** | Complex | 0.0 | 0.9 | 0.0002 |
| **Micrococcaceae** | Complex | 0.0 | 0.9 | 0.0001 |
| **Arthrobacter** | Complex | 0.0 | 0.9 | 0.0009 |
| **Flavobacteriaceae** | Complex | 0.0 | 0.8 | 0.002 |
| **Olivibacter** | Complex | 0.0 | 0.8 | 0.0002 |
| **Sphingobacteriaceae** | Complex | 0.0 | 0.8 | 0.0002 |
| **Micrococcales** | Complex | 0.0 | 0.8 | 0.0014 |
| **Sanguibacter** | Complex | 0.0 | 0.8 | 0.004 |
| **Planococcus** | Complex | 0.0 | 0.7 | 0.0023 |
| **Micrococcus** | Complex | 0.0 | 0.7 | 0.0007 |
| **Kurthia** | Complex | 0.0 | 0.7 | 0.002 |
| **Chryseobacterium** | Complex | 0.0 | 0.7 | 0.0053 |
| **Sphingobacterium** | Complex | 0.0 | 0.7 | 0.0052 |
| **Brevibacterium** | Complex | 0.0 | 0.7 | 0.0165 |
| **Rothia** | Complex | 0.0 | 0.7 | 0.001 |
| **Janibacter** | Complex | 0.0 | 0.6 | 0.0066 |
| **Dyadobacter** | Complex | 0.0 | 0.5 | 0.0282 |
| **Actinomyces** | Complex | 0.0 | 0.4 | 0.0127 |
| **Rhodococcus** | Complex | 0.0 | 0.4 | 0.0443 |
